# Mortality and Cardiovascular Outcomes Associated with Statin Use in Dialysis Patients with Atherosclerotic Cardiovascular Disease: A Systematic Review and Meta-Analysis

**DOI:** 10.1159/kbr/adjag006

**Published:** 2026-09-05

**Authors:** Kittiphan Chienwichai, Pannawat Mongkolrattanakul, Arunchai Chang, Sorawat Sangkaew

**Affiliations:** a Division of Nephrology, Department of Internal Medicine, Hatyai Hospital, Songkhla, Thailand; b Division of Nephrology, Department of Internal Medicine, Phanatnikhom Hospital, Chonburi, Thailand; c Division of Gastroenterology, Department of Internal Medicine, Hatyai Hospital, Songkhla, Thailand; d Department of Social Medicine, Hatyai Hospital, Songkhla, Thailand

**Keywords:** Atherosclerosis, HMG-CoA reductase inhibitors, End-stage renal disease, Hemodialysis, Lipid-lowering therapy

## Abstract

**Introduction:**

Dialysis patients with established atherosclerotic cardiovascular disease (ASCVD) face exceptionally high risks of mortality and recurrent cardiovascular events yet remain substantially underrepresented in major statin trials. We aimed to synthesize evidence from studies restricted to this population to evaluate the association between statin therapy and clinical outcomes.

**Methods:**

We systematically searched Embase, MEDLINE, Web of Science, Scopus, and CENTRAL databases from inception through February 20, 2025, for randomized controlled trials and observational studies evaluating statin therapy in adults receiving maintenance dialysis with documented ASCVD. Hazard ratios were pooled using random-effects models. Risk of bias was assessed using the ROBINS-I tool. The primary outcome was all-cause mortality.

**Results:**

Fourteen cohort studies comprising 242,118 patients were included in the meta-analysis. Statin therapy was associated with a significant reduction in all-cause mortality (HR 0.85, 95% CI: 0.74–0.98; *p* = 0.027; *I*^2^ = 53.1%). Associations with cardiac mortality (HR 0.88, 95% CI: 0.66–1.16; *I*^2^ = 79.6%) and major adverse cardiovascular events (HR 0.85, 95% CI: 0.59–1.21; *I*^2^ = 61.8%) were not statistically significant. Leave-one-out sensitivity analyses supported the robustness of the primary finding. Among dialysis patients with established ASCVD, the proportion of statin-treated patients was 35% and increased over time.

**Conclusion:**

Among dialysis patients with established ASCVD, statin therapy is associated with lower all-cause mortality; however, its effect on cardiovascular outcomes remains uncertain. Substantial heterogeneity and a predominance of observational evidence limit causal inference. Well-designed prospective trials in this high-risk population are warranted.

## Plain Language Summary

Patients who rely on dialysis – a treatment that filters the blood when the kidneys can no longer do so – are at very high risk of heart attacks, strokes, and death. Statins are cholesterol-lowering medicines widely used to prevent repeat heart and blood vessel problems in the general population. However, whether statins help dialysis patients who have already experienced such problems remains unclear because the major clinical trials in dialysis populations included very few patients with a prior heart attack or stroke, and current guidelines do not clearly advise whether statins should be used in this group. To address this question, we searched medical databases for all available studies examining statin use specifically in dialysis patients with a history of heart or blood vessel disease. We found 14 studies involving more than 240,000 patients. When the results were combined, dialysis patients who took statins had a 15% lower risk of dying from any cause compared with those who did not take statins. However, statins did not clearly reduce the risk of dying specifically from heart disease or of having another heart attack or stroke. The strength of the evidence was limited because all included studies were observational, meaning patients were not randomly assigned to receive statins or not, and there were notable differences between studies.

## Introduction

Patients receiving maintenance dialysis have markedly higher all-cause and cardiovascular mortality and a substantially greater burden of atherosclerotic cardiovascular disease (ASCVD) than the general population [1, 2]. Cardiovascular mortality in this population is estimated to be 10–20 times higher [3], and the presence of ASCVD confers an even greater risk of death than in patients without ASCVD [4]. Overall, ASCVD accounts for nearly half of all deaths among patients receiving dialysis [5, 6]. Furthermore, patients on dialysis who survive a first nonfatal myocardial infarction (MI) face a 144% higher risk of recurrent MI [7], underscoring persistently elevated cardiovascular morbidity.

Statins are well established for secondary prevention of ASCVD in the general population and in individuals with non-dialysis-dependent chronic kidney disease [8]. However, their benefits may not directly apply to patients who require dialysis. The two major randomized controlled trials of statins in this population, the Die Deutsche Diabetes Dialyse Studie (4D), which randomized 1,255 hemodialysis patients with type 2 diabetes to atorvastatin 20 mg or placebo [9], and AURORA, which randomized 2,776 maintenance hemodialysis patients to rosuvastatin 10 mg or placebo, each found no significant reduction in the primary cardiovascular endpoint [10]. Although 30–40% of participants in the 4D and AURORA trials had a history of cardiovascular disease, neither trial selected participants on the basis of established ASCVD, nor was either trial powered for a secondary-prevention analysis [9, 10]. Similarly, an individual participant data meta-analysis by the Cholesterol Treatment Trialists’ Collaboration, which included a relatively small subgroup of dialysis patients (65% of whom had no preexisting vascular disease), also found no significant reduction in major vascular events with statin therapy [8]. Nonetheless, several recent cohort studies using real-world data have suggested a potential survival benefit of statin therapy for secondary prevention in this population [11–14].

Current clinical guidelines provide limited or no specific recommendations regarding statin use for secondary prevention in dialysis patients with established ASCVD [15–17]. This gap in guidance highlights the ongoing uncertainty about the role of statin therapy in this high-risk population, particularly given the limited data focused specifically on patients receiving dialysis with established ASCVD. In parallel, the extent to which statins are actually prescribed in this population remains poorly characterized, and quantifying real-world prescribing patterns is essential to identify any implementation gap between evidence and practice. Accordingly, the objectives of this study were (1) to evaluate the association of statin therapy with all-cause mortality, cardiac mortality, and recurrent ASCVD events in patients receiving dialysis with established ASCVD and (2) to determine the proportion of statin-treated patients among dialysis patients with established ASCVD.

## Methods

This study is registered at PROSPERO (CRD420250611611) and follows the guidelines of Preferred Reporting Items for Systematic Reviews and Meta-Analyses (PRISMA) (online suppl. Material 1; for all online suppl. material, see https://doi.org/10.1159/kbr/adjag006) [18].

### Search Strategy

Electronic searches were conducted in Embase, MEDLINE, Web of Science, Scopus, and CENTRAL databases from their inception through February 20, 2025. Our search strategy aimed to identify RCTs and prospective or retrospective cohort studies evaluating statin therapy for secondary prevention in patients receiving dialysis with established ASCVD. Eligible studies included adult patients (≥18 years old) on dialysis with documented histories of MI, coronary artery disease (CAD), stroke, or peripheral artery disease (PAD); no upper age limit was applied. Studies were excluded if they involved pediatric populations, patients without confirmed ASCVD, focused primarily on other comorbidities without clear ASCVD outcomes, were cross-sectional, or involved non-human subjects. We combined Medical Subject Headings (MeSH) terms and free-text keywords for two concepts: dialysis (e.g., hemodialysis, peritoneal dialysis) and statins (e.g., HMG-CoA reductase inhibitors). Search syntax was adapted to each database’s specific requirements, while maintaining the core conceptual groupings. Full search strategies are detailed in online supplementary Table 1. The search was restricted to original articles written in English (books, conference abstracts, comments, notes, and letters were excluded). In addition, hand searches were conducted among the reference lists of eligible primary reports and relevant review articles.

### Article Selection

Two investigators (K.C. and P.M.) independently screened search results using Rayyan software [19]. Any discrepancies in the screening process were initially resolved through discussion between the two reviewers. If an agreement could not be reached, a third reviewer (S.S.) was consulted to make the final decision. Following the initial screening, K.C. and P.M. conducted a full-text screening to assess study eligibility. To prevent duplication, studies with overlapping cohorts were excluded. In cases of overlapping cohorts, the study with the largest sample size or longest follow-up was retained. Data extraction was performed independently by two reviewers (K.C. and P.M.) using a pre-constructed extraction form (online suppl. Material 2). The primary endpoint of our analysis was all-cause mortality at the longest available follow-up. Secondary endpoints included cardiac mortality, major adverse cardiovascular events (MACEs), nonfatal MI, nonfatal stroke, and the proportion of statin-treated patients among dialysis patients with established ASCVD. MACE was defined as a composite outcome comprising cardiac death, nonfatal MI, and nonfatal stroke.

### Internal Validity and Risk-of-Bias Assessment

We assessed nonrandomized studies using the ROBINS-I tool [20], which evaluates bias relative to a target trial across seven domains: bias due to confounding; bias in the selection of participants into the study; bias in classification of interventions; bias due to deviations from intended interventions; bias due to missing data; bias in measurement of outcomes; and bias in the selection of the reported result. Two reviewers (K.C. and P.M.) independently assessed studies using the tool’s signaling questions; disagreements were resolved through discussion and consensus, with a third reviewer (S.S.) serving as an arbiter.

### Statistical Methods

The effect measure was the hazard ratio (HR). For each study, the most fully adjusted HR and its 95% confidence interval (CI) were extracted. Analyses were performed on the natural log scale using the generic inverse-variance approach. Pooled effects were exponentiated and reported as HRs with 95% CIs. Forest plots display individual and pooled estimates. Random-effects models were fitted using the Paule-Mandel estimator [21] for between-study variance with Hartung-Knapp adjustments [22] for CIs.

Heterogeneity was assessed using the I^2^ statistic, with values above 50% indicating substantial heterogeneity, consistent with the Cochrane Handbook for Systematic Reviews of Interventions [23]. Meta-regression assessed the proportion of statin-treated dialysis patients with established ASCVD, using the median recruitment year as a moderator to examine temporal trends in prescribing patterns. We performed sensitivity analyses using a leave-one-out approach for outcomes with three or more contributing studies. This approach assessed the robustness of pooled effects and the influence of individual studies on heterogeneity. Planned subgroup analyses (e.g., by statin intensity, dialysis modality, or comorbidities) were not conducted because most studies did not report results stratified by these covariates.

Publication bias was evaluated by visually inspecting funnel plots. Egger’s test was performed on study outcomes from 10 or more studies as smaller studies limit the test’s reliability. The GRADE (Grading of Recommendations, Assessment, Development and Evaluation) approach was used to assess the certainty of the evidence for each outcome. All analyses were performed using R software (version 4.4.1) [24] with the meta [25] and metafor packages [26].

## Results

We retrieved 3,167 records. After removal of 901 duplicates, 2,266 records were screened, and 2,188 were excluded, with reasons detailed in online supplementary Table 2. Of 78 reports assessed in full, 14 studies were included in the review (*N* = 242,118; Fig. 1) [12, 14, 27–38]. The characteristics of the included studies are shown in Table 1, and detailed baseline characteristics of the patients are presented in Table 2. No randomized controlled trial was included as none met our eligibility criteria. The median sample size was 533 (range, 93–197,716). Follow-up durations varied considerably and were reported using different summary measures: the maximum follow-up in 5 studies [12, 14, 27, 28, 32], the mean follow-up in 5 studies [29–31, 36, 37], and the median follow-up in 4 studies [33–35, 38].

**Fig. 1.**
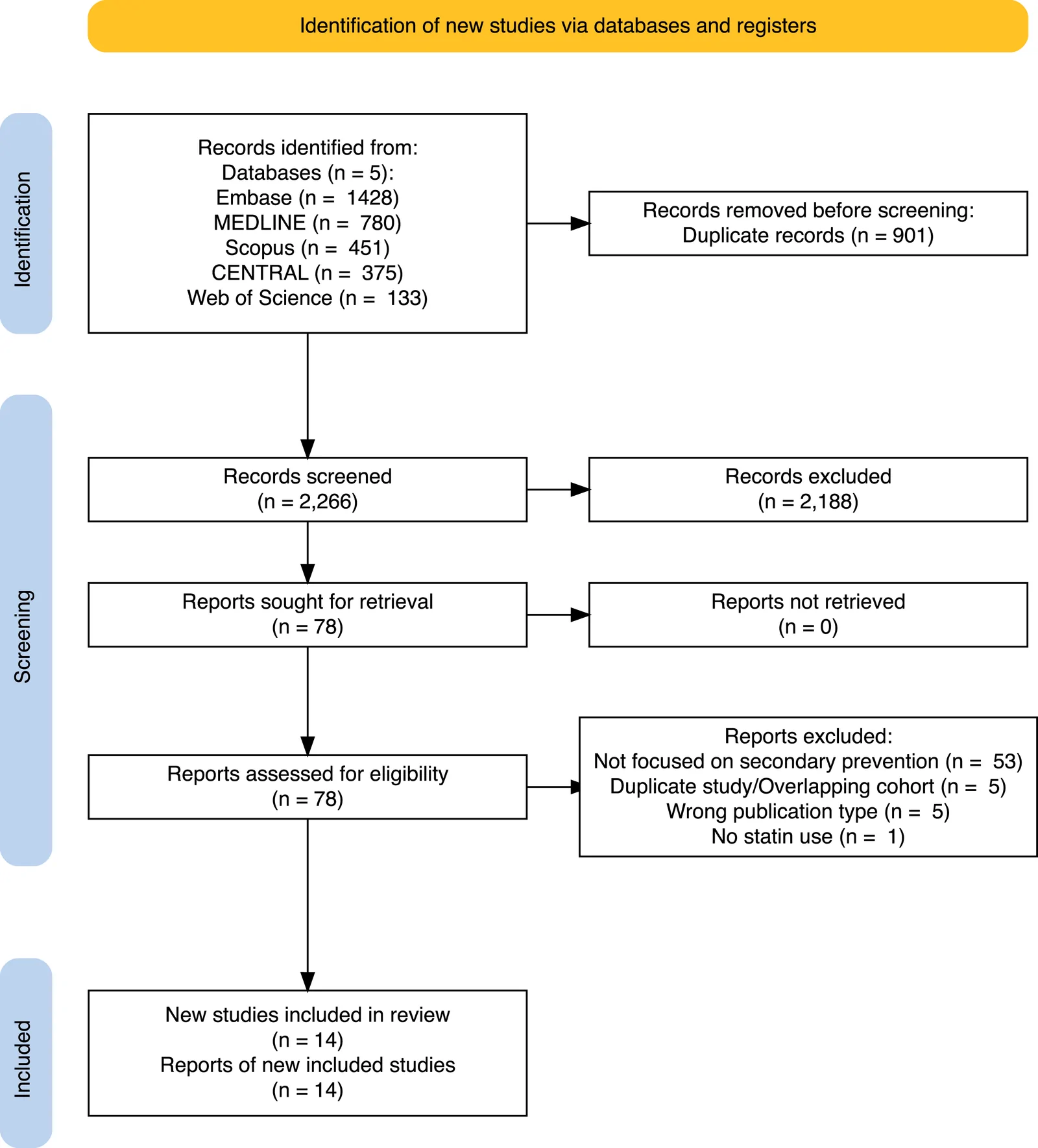
PRISMA flow diagram of study selection. This diagram illustrates the systematic process of identifying, screening, evaluating, and including studies for the meta-analysis, in accordance with PRISMA guidelines.

**Table 1.** Characteristics of studies included in the systematic review and meta-analysis

Author	Year	Setting	Country	Design	Sample size	Statin, *n* (%)	No statin, *n* (%)	Dialysis modality	Type of ASCVD	Recruitment period	Follow-up
Mason et al. [27]	2005	Multicenter	France, Germany, Italy, Spain, UK, Japan, USA	Retrospective cohort	2,460	431 (17.5)	2,029 (82.5)	HD only	CAD	1996–1999	Up to 5 years
Winkelmayer et al. [28]	2006	Population-based	USA	Retrospective cohort	494	96 (19.4)	398 (80.6)	HD and PD	AMI	1995–2003	Up to 1 year after the index date
Natsuaki et al. [38]	2012	Multicenter	Japan	Retrospective cohort	572	117 (20.5)	455 (79.5)	HD only	Undergoing PCI or CABG	2005–2007	Median 956 (699–1,245) days
Kagawa et al. [29]	2014	Single center	Japan	Retrospective cohort	96	16 (16.7)	80 (83.3)	HD and PD	CAD	2004–2006	Mean 4.9±1 years
Aftab et al. [30]	2015	Single center	USA	Prospective cohort	127	104 (81.9)	23 (18.1)	HD only	CAD	2008	Up to 2.0 years
Chung et al. [14]	2017	Population-based	Taiwan	Retrospective cohort	3,910	847 (21.7)	3,063 (78.3)	HD and PD	AMI	2001–2013	Up to 4 years
Kim et al. [31]	2018	Population-based	South Korea	Retrospective cohort	150	113 (75.3)	37 (24.7)	HD only	Undergoing PCI	2002–2013	Mean 3.15±2.71 years
Hu et al. [32]	2020	Single center	Taiwan	Retrospective cohort	93	34 (36.6)	59 (63.4)	HD and PD	Undergoing CABG	2005–2016	Mean 4.09±3.11 years
Kuo et al. [12]	2021	Population-based	Taiwan	Retrospective cohort	8,056	2,134 (26.5)	5,922 (73.5)	HD and PD	AMI	2000–2012	Up to 12 years
Shavadia et al. [33]	2021	Population-based	USA	Retrospective cohort	197,716	47,562 (24.1)	150,154 (75.9)	HD and PD	CAD	2006–2014	Median of 622 (299–1,224) days
Funamizu et al. [34]	2022	Single center	Japan	Retrospective cohort	201	73 (36.3)	128 (63.7)	HD only	Undergoing PCI	2000–2016	Median 2.8 (0–15.2) years
Horikoshi et al. [35]	2022	Multicenter	Japan	Prospective cohort	234	91 (38.9)	143 (61.1)	HD only	Undergoing PCI	2008–2018	Median 30 (13–36) months
Lo et al. [37]	2022	Population-based	Taiwan	Retrospective cohort	10,767	3,597 (33.4)	7,170 (66.6)	HD and PD	PAD	2001–2013	Mean 3.0±2.4 years
Lee et al. [36]	2023	Population-based	South Korea	Retrospective cohort	17,242	9,611 (55.7)	7,631 (44.3)	HD and PD	CAD, CVA, or PAD (composite outcome)	2013–2018	Mean 32.6±20.9 months

AMI, acute myocardial infarction; ASCVD, atherosclerotic cardiovascular disease; CABG, coronary artery bypass grafting; CAD, coronary artery disease; CVA, cerebrovascular accident; HD, hemodialysis; IQR, interquartile range; NR, not reported; PAD, peripheral artery disease; PCI, percutaneous coronary intervention; PD, peritoneal dialysis.

**Table 2.** Baseline characteristics of patients included in the meta-analysis

Author	Age		Male, *n* (%)		Hypertension, *n* (%)		Diabetes mellitus, *n* (%)		Heart failure, *n* (%)		LDL level, mg/dL		ACEi/ARB, *n* (%)		Beta-blocker, *n* (%)		Aspirin, *n* (%)	
	statin	no statin	statin	no statin	statin	no statin	statin	no statin	statin	no statin	statin	no statin	statin	no statin	statin	no statin	statin	no statin
Winkelmayer^a^ et al. [28]	75.6±6.3		171 (34.6)		380 (76.9)		354 (71.7)		395 (80.2)		–		222 (44.9)		145 (29.4)		–	
Natsuaki et al. [38]	65.6±11.0	65.5±9.7	68 (58)	363 (80)	105 (90)	392 (86)	72 (62)	274 (60)	43 (37)	144 (32)	102±35.1	94.4±29.6	63 (54)	198 (44)	43 (37)	100 (22)	115 (98)	444 (98)
Kagawa et al. [29]	64.7±6.7	67.8±10.8	9 (56.3)	61 (76.3)	16 (100)	66 (82.5)	13 (81.2)	46 (57.5)	7 (43.8)	39 (48.8)	111.5±51.5	99.3±30.0	7 (43.8)	39 (48.8)	8 (50.0)	20 (25.0)	16 (100)	78 (97.5)
Chung et al. [14]	66.0±11.5	66.6±11.5	458 (58.0)	445 (56.3)	533 (67.5)	546 (69.1)	364 (46.1)	370 (46.8)	359 (45.4)	370 (46.8)	–	–	425 (53.8)	452 (57.2)	457 (57.8)	450 (57.0)	551 (69.7)	562 (71.1)
Kim et al. [31]	Median 67	Median 67	65 (57.5)	20 (54.1)	110 (97.3)	37 (100.0)	95 (84.1)	32 (86.5)	–	–	–	–	–	–	–	–	–	–
Hu et al. [32]	58.7±9.6	62.0±10.3	26 (76.5)	47 (79.7)	–	–	24 (70.6)	40 (67.8)	–	–	–	–	–	–	–	–	–	–
Kuo et al. [12]	65.7±11.3	67.8±11.4	1,122 (52.6)	3,219 (54.3)	2,035 (95.4)	5,417 (91.5)	1,556 (72.9)	4,045 (68.3)	1,022 (47.9)	2,669 (45.1)	–	–	1,486 (69.6)	2,683 (45.3)	1,461 (68.5)	2,620 (44.2)	1,914 (89.7)	3,849 (65.0)
Shavadia et al. [33]	Median 69	Median 65	23,020 (48.4)	78,531 (52.3)	43,091 (90.6)	133,637 (89.0)	27,066 (56.8)	69,071 (46.0)	–	–	–	–	14,839 (31.2)	22,974 (15.3)	24,590 (51.7)	36,037 (24.0)	–	–
Funamizu et al. [34]	65.2±11.9	66.6±9.3	56 (76.7)	112 (87.5)	60 (82.2)	114 (89.1)	–	–	–	–	89.6±27.3	101.0±31.3	33 (45.2)	73 (57.0)	39 (53.4)	57 (44.5)	–	–
Horikoshi^a^ et al. [35]	Median 68 (62–74)		173 (73.9)		193 (82.5)		146 (62.4)		234 (100)		90 (70–115)		103 (44.0)		92 (39.3)		–	
Lo et al. [37]	65.8±11.4	68.5±11.5	1,698 (47.2)	3,476 (48.5)	3,406 (94.7)	6,601 (92.1)	3,052 (84.8)	5,555 (77.5)	–	–	–	–	2,129 (59.2)	3,427 (47.8)	2,256 (62.7)	3,604 (50.3)	1,968 (54.7)	2,846 (39.7)
Lee et al. [36]	63.2±11.0	62.8±11.7	6,016 (62.6)	4,634 (60.7)	9,066 (94.3)	7,075 (92.7)	7,004 (72.9)	4,896 (64.2)	4,771 (49.6)	3,177 (41.6)	–	–	–	–	–	–	–	–

Data are presented as mean ± SD, median (interquartile range), or *n* (%) unless otherwise specified.

ACEi, angiotensin-converting enzyme inhibitor; ARB, angiotensin receptor blocker; IQR, interquartile range; LDL, low-density lipoprotein.

^a^ Characteristics reported for the entire cohort.

Note: Baseline characteristics were not separately reported for the CAD subgroup in the studies by Mason et al. [27] and Aftab et al. [30].

Six studies were conducted in 3,744 patients receiving hemodialysis [27, 30, 31, 34, 35, 38] and eight in 238,374 patients receiving hemodialysis or peritoneal dialysis [12, 14, 28, 29, 32, 33, 36, 37]. Twelve studies were conducted in patients with CAD [27, 29, 30, 33], MI [12, 14, 28], or undergoing coronary-related procedures (percutaneous coronary intervention or coronary artery bypass grafting) [31, 32, 34, 35, 38]. One study focused specifically on PAD [37], and another included a composite population with CAD, cerebrovascular accident, or PAD [36]. The included studies were conducted across various countries, predominantly in Asia (e.g., Taiwan, Japan, South Korea) and North America (the USA). Five studies were excluded due to overlapping cohorts; details are provided in online supplementary Table 3.

### Risk of Bias

Risk-of-bias assessments are presented in online supplementary Figures 1 and 2. All 14 studies were judged to have an overall moderate risk of bias, driven primarily by moderate risk of confounding across all studies and moderate risk in classification of interventions in 11 studies. The remaining domains were predominantly rated as low risk.

### Study Outcomes

#### All-Cause Mortality

In ten studies comprising 125,909 participants, statin therapy was associated with a significantly lower risk of all-cause mortality compared with control (HR = 0.85, 95% CI: 0.74–0.98, *p* = 0.027; Table 3; Fig. 2). Substantial heterogeneity was observed (*I*^2^ = 53.1%), and the certainty of evidence was rated as low, as detailed in the GRADE quality assessment (online suppl. Table 4). Leave-one-out influence analyses demonstrated that the pooled HR remained consistent, ranging from 0.83 to 0.89 across iterations (online suppl. Table 5).

**Table 3.** Summary of primary and secondary outcomes

Outcome analyzed	No. of studies reporting outcome	No. of statin patients	No. of non-statin users	HR (95% CI)	*p* value for Effect	*I* ^2^ (95% CI)	Certainty of evidence (GRADE)
All-cause mortality	10	61,904	64,005	0.85 (0.74–0.98)	0.027	53.1%	⊕⊕⊝⊝ Low
Cardiac mortality	5	14,870	17,260	0.88 (0.66–1.16)	0.262	79.6%	⊕⊝⊝⊝ Very low
MACE	5	49,724	50,041	0.85 (0.59–1.21)	0.263	61.8%	⊕⊝⊝⊝ Very low
MI	2	4,025	4,025	1.12 (0.48–2.59)	0.334	0.0%	⊕⊝⊝⊝ Very low
Stroke	2	4,025	4,025	1.12 (0.84–1.50)	0.128	0.0%	⊕⊝⊝⊝ Very low

GRADE Working Group grades of evidence: high certainty: we are very confident that the true effect lies close to that of the estimate of the effect. Moderate certainty: we are moderately confident in the effect estimate: the true effect is likely to be close to the estimate of the effect, but there is a possibility that it is substantially different. Low certainty: our confidence in the effect estimate is limited: the true effect may be substantially different from the estimate of the effect. Very low certainty: we have very little confidence in the effect estimate: the true effect is likely to be substantially different from the estimate of effect.

MACE, major adverse cardiovascular event.

**Fig. 2.**
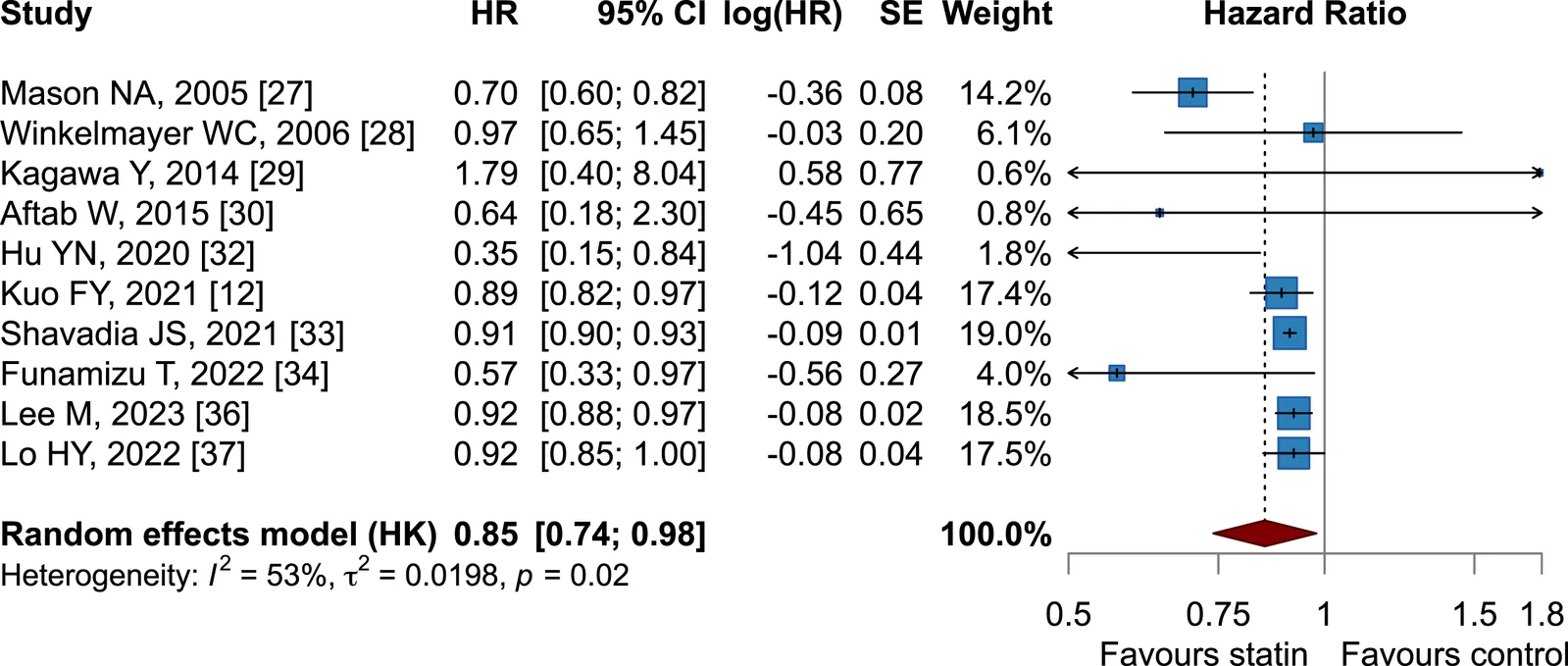
Forest plot of all-cause mortality. This plot displays the hazard ratios (HRs) and 95% confidence intervals (CIs) for all-cause mortality from the individual studies included in the meta-analysis, along with the pooled estimate from the random-effects model.

#### Cardiac Mortality

A pooled analysis of five studies with 32,130 participants found no statistically significant reduction in cardiac mortality with statin use (HR = 0.88, 95% CI: 0.66–1.16, *p* = 0.262; Table 3; Fig. 3). Substantial heterogeneity was observed (*I*^2^ = 79.6%), and the certainty of evidence was rated as very low (online suppl. Table 4). Leave-one-out influence analyses showed that the pooled HR remained broadly consistent across iterations (0.84–0.92). Exclusion of Lee et al. [36] reduced heterogeneity to 0% (HR = 0.84, 95% CI: 0.73–0.97, *p* = 0.030), but no single study materially altered the direction of the association (online suppl. Table 6).

**Fig. 3.**
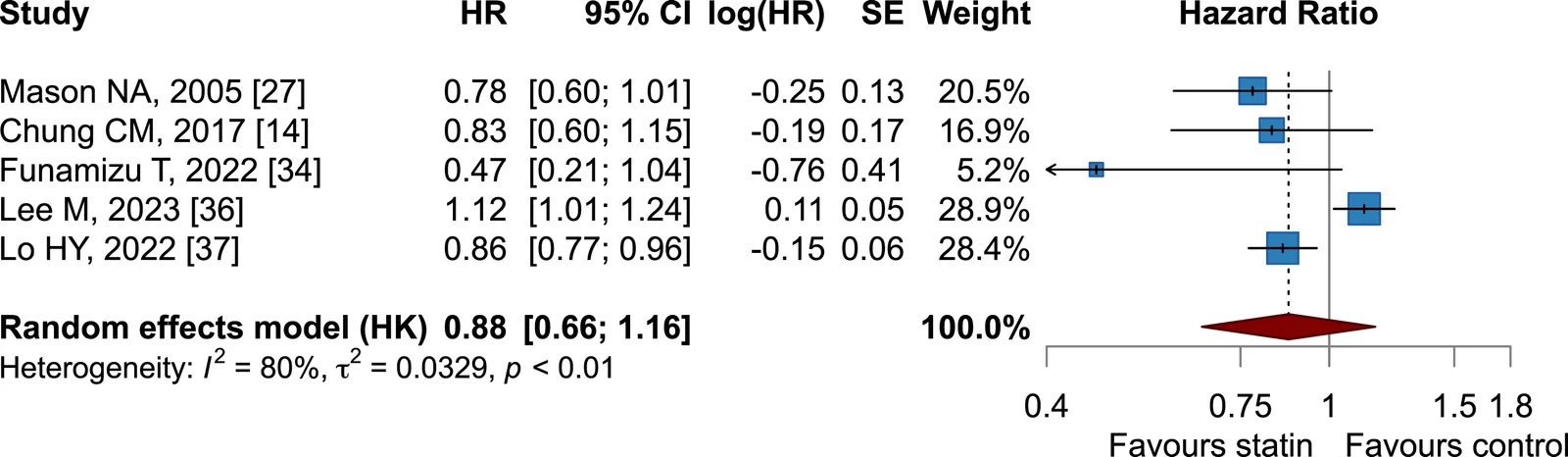
Forest plot of cardiac mortality. This plot displays the hazard ratios (HRs) and 95% confidence intervals (CIs) for cardiac mortality from the individual studies included in the meta-analysis, along with the pooled estimate from the random-effects model.

### Major Adverse Cardiovascular Events

Among five studies comprising 99,765 participants and evaluating MACE, statin use was not associated with a statistically significant reduction in risk (HR = 0.85, 95% CI: 0.59–1.21, *p* = 0.263; Table 3; Fig. 4). Substantial heterogeneity was observed (*I*^2^ = 61.8%), and the certainty of evidence was rated as very low (online suppl. Table 4). Leave-one-out influence analyses showed that the pooled HR remained consistent across iterations (online suppl. Table 7).

**Fig. 4.**
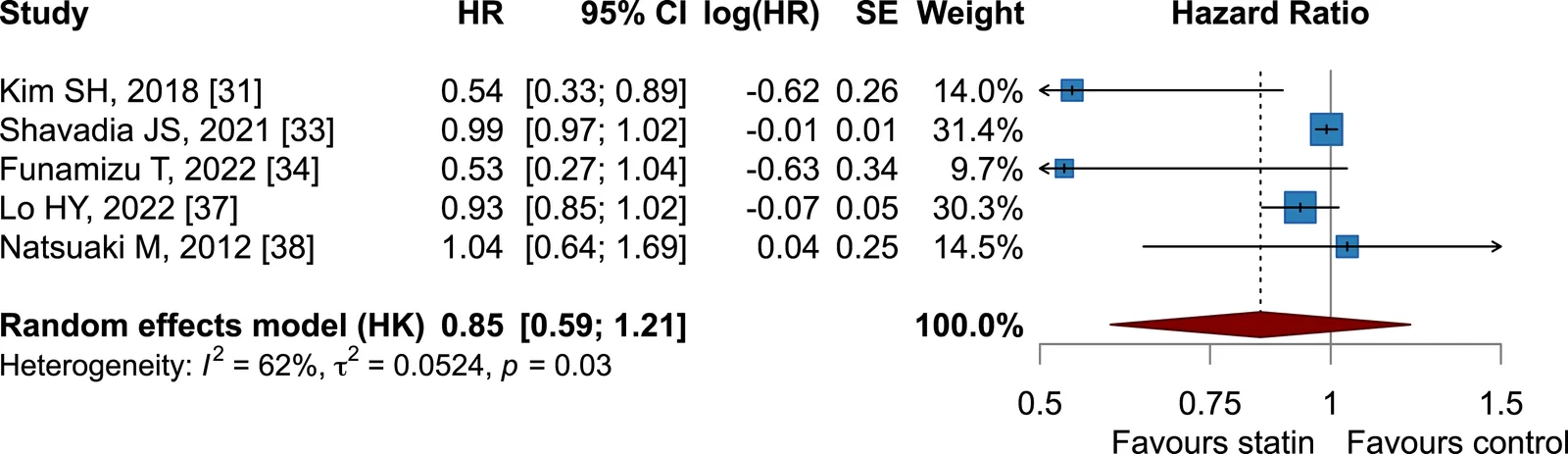
Forest plot of major adverse cardiovascular events (MACEs). This plot displays the hazard ratios (HRs) and 95% confidence intervals (CIs) for MACE from the individual studies included in the meta-analysis, along with the pooled estimate from the random-effects model.

### Myocardial Infarction

A pooled analysis of two studies comprising 8,050 participants found no statistically significant effect of statin therapy on MI risk (HR = 1.12, 95% CI: 0.48–2.59, *p* = 0.334; Table 3; online suppl. Fig. 4). No heterogeneity was observed (*I*^2^ = 0.0%), and the certainty of evidence was rated as very low (online suppl. Table 4).

### Stroke

A pooled analysis of two studies comprising 8,050 participants found no statistically significant association between statin therapy and stroke risk (HR = 1.12, 95% CI: 0.84–1.50, *p* = 0.128; Table 3; online suppl. Fig. 5). No heterogeneity was observed (*I*^2^ = 0.0%), and the certainty of evidence was rated as very low (online suppl. Table 4).

### Proportion of Statin-Treated Patients among Dialysis Patients with Established ASCVD

Across all 14 cohorts (242,118 patients), the proportion of dialysis patients with established ASCVD receiving statin therapy varied markedly, from 17% to 82%; the pooled estimate was 35% (95% CI: 27%–43%; online suppl. Fig. 6). Substantial heterogeneity was observed across studies (*I*^2^ = 99.8%). In a meta-regression of the logit-transformed proportion of statin use, the median recruitment year was associated with higher statin use (OR 1.10 per year, 95% CI: 1.03–1.18; *p* = 0.003) (Fig. 5). This temporal model explained 35.0% of the between-study heterogeneity.

**Fig. 5.**
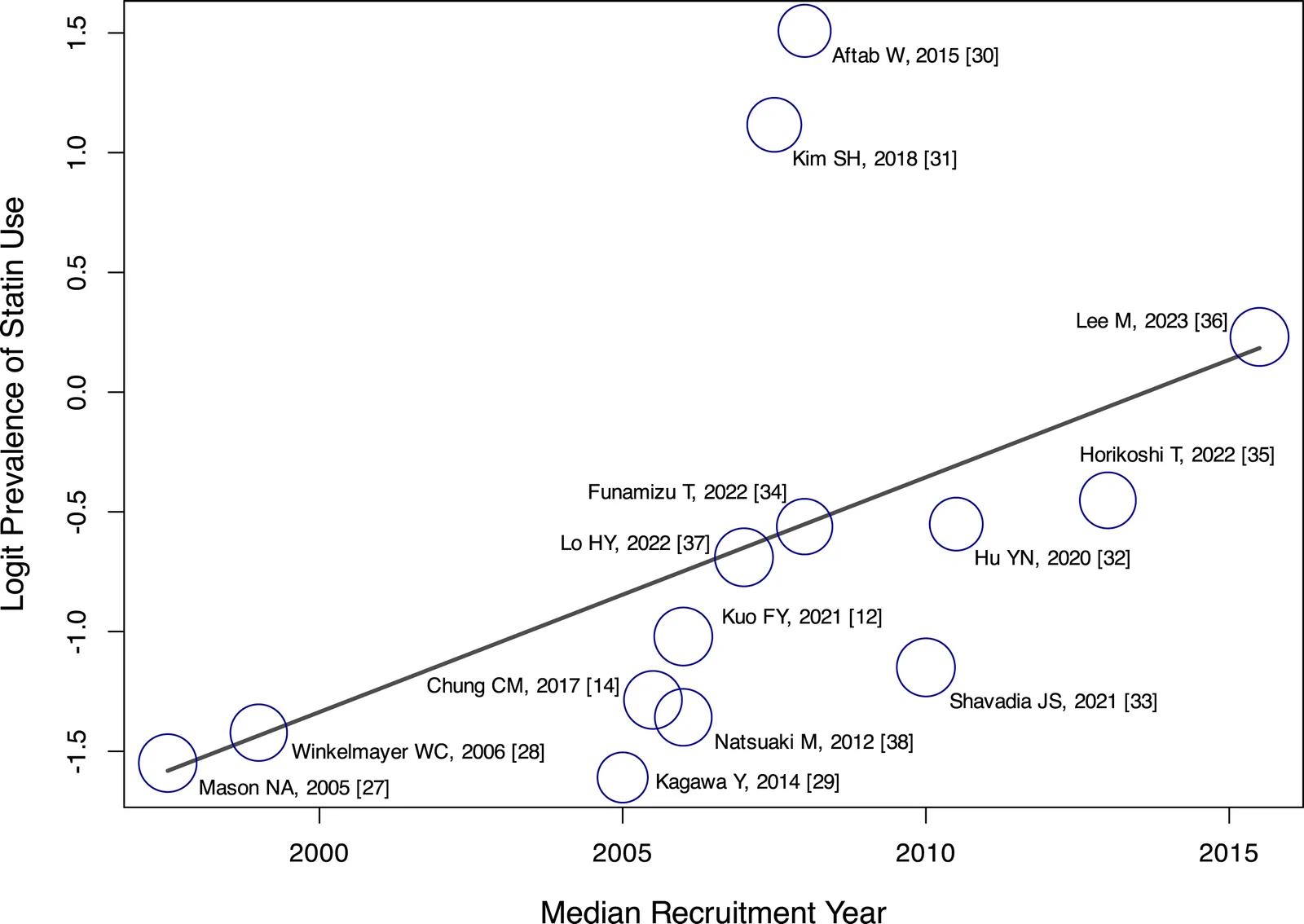
Meta-regression plot of proportion of statin-treated patients among dialysis patients with established ASCVD over time. This plot illustrates the relationship between the median recruitment year of the studies and the proportion of statin-treated patients among dialysis patients with established ASCVD. Each point represents an individual study, with the point’s size reflecting the study’s weight.

### Publication Bias

Funnel plot inspection did not suggest substantial asymmetry for any outcome (online suppl. Fig. 3A–F). Egger’s regression test for all-cause mortality (10 studies) did not yield a statistically significant result (*p* = 0.352). For cardiac mortality, MACE, MI, and stroke, the small number of contributing studies (2–5 per outcome) precluded formal testing, and funnel plots for these outcomes should be interpreted with caution.

## Discussion

This study provides the most comprehensive evidence synthesis to date on statin therapy for secondary prevention in dialysis patients with ASCVD, drawing on 14 studies and over 240,000 patients. Statin use was associated with reduced all-cause mortality but showed no significant effect on cardiac mortality, MACE, MI, or stroke. Sensitivity analyses confirmed the robustness of these findings across outcomes. Notably, for cardiac mortality, excluding one influential study eliminated heterogeneity and revealed a significant protective association with statins. Using GRADE methodology, we rated the certainty of evidence as low for all-cause mortality and very low for all other outcomes. Additionally, the pooled proportion of statin-treated patients among dialysis patients with established ASCVD was only 35%, despite evidence of a significant temporal increase over the past 2 decades.

Previous randomized trials [9, 10] and meta-analyses [8] in dialysis populations enrolled few patients with established ASCVD and were not designed for secondary prevention. By focusing on patients with documented ASCVD, this review addresses a major evidence gap and provides estimates more relevant to secondary prevention in dialysis. The observed reduction in all-cause mortality is directionally consistent with the benefits of statins outside the dialysis setting, although cardiovascular-specific endpoints remain neutral in the primary analysis.

The observed mortality benefit of statins in our study may be attributed to two main factors. First, statin effects in dialysis appear heterogeneous, with specific patient subgroups likely to derive greater benefit [39]. This is supported by post hoc analysis of RCTs, which have indicated that dialysis patients at higher cardiovascular risk, such as those with diabetes [40], elevated LDL cholesterol [41], or a high burden of atherosclerosis-related risk factors [39], may indeed benefit from statin therapy. Second, because infection is also a leading cause of death in this population [5], the pleiotropic effects of statins, including immunomodulatory and anti-inflammatory actions, may contribute to improved survival from infectious diseases [42]. This hypothesis is supported by evidence from non-dialysis populations, where statins have demonstrated protective effects against infections [43–45]. These immunomodulatory benefits of statins may explain why our analysis observed a significant reduction in all-cause mortality but not in cardiovascular-specific mortality.

The absence of significant reductions in cardiovascular mortality and MACE in our primary analysis contrasts with the statin benefits observed in non-dialysis populations [46, 47]. A likely explanation lies in the distinct pattern of cardiovascular death among dialysis patients. A large proportion of cardiovascular mortality in this population stems from sudden cardiac death and heart failure rather than atherosclerotic events [48]. These modes of death are driven by processes characteristic of the dialysis population, including vascular calcification [49], low dialysate calcium and potassium [50], aggressive fluid removal [51], and left ventricular hypertrophy with diffuse myocardial fibrosis [52]. These factors are largely independent of atherothrombosis and therefore unlikely to be modifiable by statin therapy. Competing risks may further obscure any cardiovascular-specific benefit. Dialysis patients carry a high burden of non-cardiovascular mortality from infection, volume overload, and electrolyte disturbances; when such competing events are frequent, cause-specific hazard models that treat non-cardiovascular deaths as non-informative censoring can underestimate the cumulative incidence of cardiovascular events, potentially masking a genuine reduction in atherosclerotic disease.

Aside from statins, therapies proven beneficial in the general population have not been shown to improve cardiovascular outcomes. Spironolactone did not reduce the composite of cardiovascular mortality or hospitalization for heart failure in the ACHIEVE trial [53], and low-dose rivaroxaban did not reduce MACEs but increased major bleeding in the TRACK trial [54]. This convergence of neutral results across lipid-lowering, mineralocorticoid receptor antagonism, and anticoagulation suggests that the drivers of cardiovascular mortality in dialysis patients differ from those in the general population and underscores the need for further research into both the pathophysiology and treatment of cardiovascular disease in this group.

Leave-one-out influence analysis confirmed the robustness of the all-cause mortality estimate, with the pooled HR remaining stable across leave-one-out iterations. For cardiac mortality, excluding Lee et al. [36] reduced heterogeneity from 79.6% to 0% and yielded a significant pooled estimate (HR 0.84, 95% CI: 0.73–0.97). This study was the only one in which statin therapy was associated with higher cardiac mortality (adjusted HR 1.12, 95% CI: 1.01–1.23), likely reflecting its inclusion of a broader ASCVD population (coronary heart disease, cerebrovascular accident, and PAD) compared with the exclusively coronary populations in other studies, along with a more limited covariate adjustment strategy without propensity score methods. These findings suggest that clinical and methodological heterogeneity, rather than the absence of a true effect, may partly explain the neutral primary results for cardiovascular endpoints.

Heterogeneity was substantial and likely multifactorial, reflecting differences in dialysis modality, statin class and intensity, exposure definition and adherence, outcome definitions and ascertainment, follow-up duration, baseline cardiovascular risk, and regional practice patterns. Subgroup analyses were limited by incomplete and inconsistent reporting, precluding robust evaluation of dose-response relationships and modality-specific effects.

The predominantly moderate risk of bias across studies reflects inherent limitations of observational designs. Residual confounding is the most pervasive concern as patients prescribed statins after an ASCVD event may be systematically healthier, more adherent, and more likely to receive comprehensive secondary prevention. Most studies also carried a moderate risk of exposure classification because statin use was typically assessed at hospital discharge without accounting for subsequent changes in use or adherence during follow-up. While anchoring exposure at discharge mitigates immortal-time bias, it does not capture treatment dynamics over time, potentially leading to misclassification of long-term exposure status. These biases generally favor statin users and may partly explain the observed reduction in all-cause mortality.

Compared with the only other meta-analysis specifically examining this population [55], our study advances the evidence base through several methodological and analytical enhancements. First, a broader database search – five databases versus three – identified more eligible studies (14 vs. 10), reducing the risk of selection bias in the evidence synthesis. Second, and more importantly, we extracted effect estimates from Shavadia et al. [33]’s published corrigendum [56], whereas the analysis by Elkoumi et al. relied on the original, uncorrected publication. Because this single study contributed the largest sample in the pool (N = 197,716), using corrected data represents a substantive difference in analytical accuracy rather than a minor technical distinction. Third, beyond clinical outcomes, our study is the first to systematically quantify the pooled proportion of statin-treated patients among dialysis patients with established ASCVD, situating the efficacy findings within real-world practice patterns and revealing a significant implementation gap in secondary prevention.

Despite the high baseline risk in dialysis patients with ASCVD, statin use remained modest and varied widely between studies. The survival signal observed in this review, coupled with the underuse of statins in practice, underscores a significant implementation gap in secondary prevention for this population. This gap is not directly addressed by current guidelines, which remain cautious about initiating statins in patients undergoing dialysis. Clinical decisions should weigh individual cardiovascular risk, adverse drug effects, pill burden, and patient preference, while recognizing the low certainty of current evidence.

Key strengths of this review include its a priori focus on secondary prevention and the use of influence analyses to assess the robustness of findings. Limitations stem from the observational nature of the included cohorts, which may lead to residual confounding and exposure misclassification. We were also unable to incorporate randomized trial data as we did not have access to individual patient-level data from the trials, which would have been required to isolate the ASCVD subgroups from the broader cardiovascular disease populations reported. Additional constraints include substantial heterogeneity not fully explained by available moderators and limited statistical power for several endpoints, given the small number of contributing studies. These factors necessitate cautious interpretation and preclude firm causal inference.

## Conclusion

In dialysis patients with established ASCVD, statin therapy is associated with lower all-cause mortality, but its effects on cardiovascular-specific outcomes remain uncertain. Given the low certainty of evidence and substantial heterogeneity, definitive recommendations will require evidence from dedicated RCTs. In the meantime, clinicians should individualize statin therapy decisions, balancing potential survival benefits against possible adverse drug effects, pill burden, and alignment with patient preferences.

## Statement of Ethics

A Statement of Ethics is not applicable because this study is based exclusively on published literature.

## Conflict of Interest Statement

The authors have no conflicts of interest to declare.

## Funding Sources

This study was not supported by any sponsor or funder.

## Author Contributions

K.C. and P.M. conceived and designed the study and conducted study screening and data extraction. K.C., A.C., and S.S. performed statistical analysis and interpreted the data. K.C. drafted the manuscript. All authors critically revised the manuscript and approved the final version.

## Data Availability

The data that support the findings of this study were derived from previously published articles. Owing to copyright and licensing restrictions of the respective publishers, the compiled dataset is not publicly available. The extracted data used for the meta-analysis are available from the corresponding author upon reasonable request.
